# Comparing Xenium 5K and Visium HD data from identical tissue slide at a pathological perspective

**DOI:** 10.1186/s13046-025-03479-4

**Published:** 2025-07-26

**Authors:** Mengping Long, Taobo Hu, Weixin Wang, Junshun Gao, Nan Wang, Mats Nilsson

**Affiliations:** 1 Science for Life Laboratory, Department of Biochemistry and Biophysics, Stockholm University, Stockholm, Sweden; 2 Cosmos Wisdom Biotech Co. Ltd, Building 10th, No. 617 Jiner Road, Hangzhou, 311215 China

## Abstract

Recent advancements in spatial transcriptomics have been largely triggered by two high-resolution technologies: Visium-HD and Xenium in-situ. While sequencing-based Visium HD features a refined bin size of 2 µm and transcriptome wide coverage, Xenium in-situ is a targeted imaging-based detection technology with sub-micron resolution. Herein we use a publicly available lung dataset which contains Visium-HD and Xenium-5K data generated on identical tissue slides to make a bona-fide technical comparison aligned with thorough pathological annotations. Whilst Visium-HD offers a broader gene coverage for detection and likely detects more tumor subclones, Xenium-5K achieves comparable results when robust clustering algorithms are applied. Importantly, from the pathological point of view, the single-cell segmentation accuracy is essential when analyzing irregularly shaped cells, where Xenium may be in favor. At the opposite side, although Xenium-5K based cell segmentation to delineate immune cells, normal lung, and vasculature at cell resolution is decent, it relies on fluorescent signals for transcript detection, which is challenging in heavily pigmented tissues such as melanoma or dust-laden alveolar macrophages, an application scenario for which Visium HD may stand out. From this perspective, pathological derived factors are the prior consideration for selecting an appropriate ST approach under difference research settings including cancer.

**Supplementary Information:**

The online version contains supplementary material available at 10.1186/s13046-025-03479-4.

## Introduction

The recent releases of Visium-HD and Xenium represents an unprecedented leap in spatial transcriptomics (ST) by achieving true single-cell resolution while retaining high analytical plexity [1, 2]. With Visium-HD increasing its resolution to 2 µm, Xenium expands its total detectable genes to 5,000. However, the rationale as how to select between them remains elusive. Given the considerable cost of ST, it’s important to select the right technology for the research questions in hand. In this short communication, we compare ST data generated using Xenium-5K and Visium-HD on the identical lung tissue adding values from pathological perspective to provide novel insights and guidance for researchers within this field.

## Results

### Comparative analysis of Visium-HD and Xenium-5K reveals platform-specific clustering patterns in identical LUAD slide

**Fig. 1:**
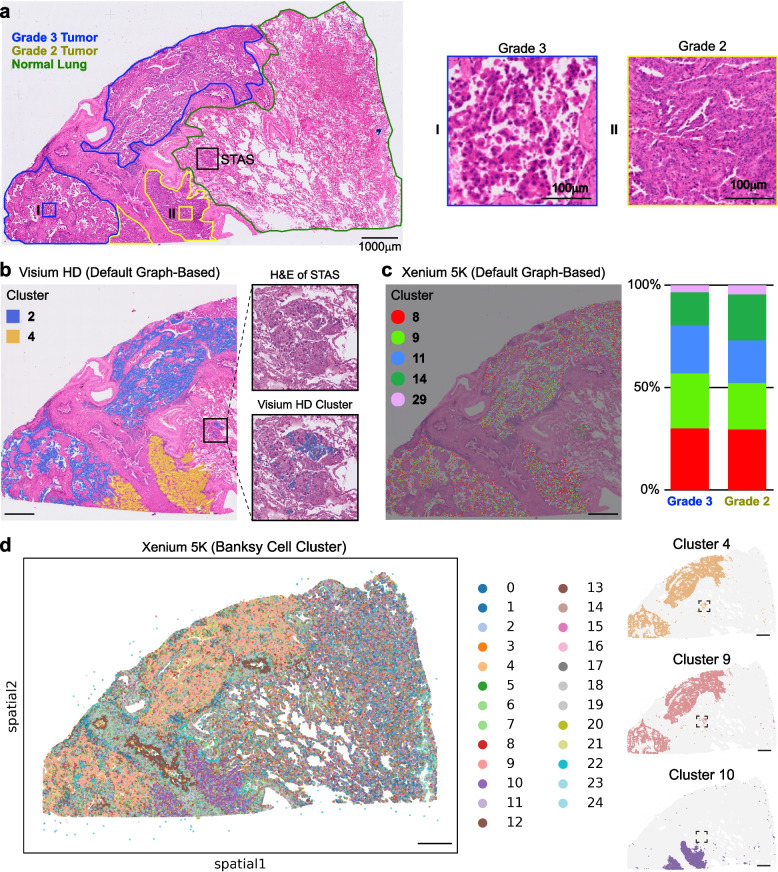
Comparison of invasive LUAD profiled by Visium-HD and Xenium-5K. **a** H&E image of the LUAD section. Pathology outlines highlight the G2 tumor region (yellow), G3 tumor region (blue), and adjacent normal lung tissue (green). A spread-through-air-spaces (STAS) focus is boxed in black; higher-magnification views are shown at right. **b** Tumor cell clusters identified by Visium-HD using 8 µm bins. Tumor cells in the G2 and G3 regions correspond to clusters 4 and 2, respectively. Enlarged STAS images reveal that the disseminated cells also map to cluster 2, indicating origin from the poorly differentiated (G3) tumor. **c** Tumor cell clusters identified by Xenium-5K. Five distinct tumor clusters are resolved across the lesion. Stacked bar plot shows that all five clusters are distributed evenly between the G2 and G3 regions. **d** Left, Xenium-5K reclustering with the Banksy algorithm: the G3 region is dominated by clusters 4 and 9, whereas the G2 region is enriched for cluster 10. Right, cells within the STAS focus belong to clusters 4 and 9, further supporting their derivation from poorly differentiated tumor cells

We downloaded Xenium-5K and Visium-HD data conducted on the same invasive lung adenocarcinoma (LUAD) and compared detection coverage on matched regions between them. Of the 18,082 genes, 4,828 (26.4%) were shared, 13,254 (72.6%) were detected in Visium HD-only, and 173 (0.9%) were Xenium-only with similar cumulative expression distribution (Supplementary Fig. 1a/c). The correlation of shared gene expression between technologies was decent (*R*^2^ = 0.64) with Xenium having twice more of the read counts compared to Visium (Supplementary Fig. 1b/c). H&E staining identified three histologically regions: a moderately differentiated area with acinar and papillary growth patterns, a poorly differentiated area exhibiting a micropapillary pattern, and adjacent normal lung tissue (Fig. 1a). A single focus of spreading through airspace (STAS) was also observed in the adjacent normal lung tissue (Fig. 1a). LUAD differentiation is pathologically defined as well (G1), moderately (G2), or poorly (G3) differentiated based on growth patterns [3, 4], correlating with different treatment strategies, prognosis, and metastatic risk. Morphologically, these correspond to lepidic (G1), acinar/papillary (G2), and micropapillary/solid/complex glandular (G3) patterns.

Using graph-based clustering segmented with 8 µm-bin in Visium-HD data, the heterogeneous tumor epithelium was grouped into two major clusters (Cluster 2 and 4 in Fig. 1b). Consistent with our pathological findings, Cluster 4 was localized within the moderately differentiated region (G2), whereas Cluster 2 corresponded to the poorly differentiated region (G3) suggesting distinct molecular characteristics underlie the pathological status of tumors by grading. These differentially expressed genes between clusters are listed in Supplementary Table 1. Interestingly, as for Xenium-5K data from the same tissue, when analyzed using the default graph-based clustering method, resulted in five tumor epithelial clusters (Clusters 8, 9, 11, 14, and 29; Fig. 1c). Unlike the Visium-HD, these clusters exhibited a rather random distribution pattern across the tumor regions (Chi squared test *p* < 0.05). We suspected the difference may potentially arise from detection principles, segmentation strategy and analytical resolution, some of which may screw the clustering algorithm robustness. To further explore, we plotted ILDR2, a top differentially expressed gene (DEG) identified from Visium-HD onto the Xenium in situ and observed a clear expression difference between G2 and G3 regions, indicating Xenium was capable of capturing relevant molecular differences. Moreover, among the 176 Xenium-only genes, ABCC6 also showed region-specific expression (Supplementary Fig. 2), further supporting our assumption. We subsequently applied the Banksy algorithm to re-analyze the Xenium data [5]. This approach resulted in clear regional separation of G2 and G3 tumor regions (Fig. 1d), underscoring that both data processing approaches and pathological information are critical for analyzing spatial transcriptomic data. While the clustering method is well documented for Visium, the default graph-based clustering for Xenium-5K has not been well described. Detailed information of all clustering methods used in this study is provided in the Methods section provided in supplementary files.

Further, to examine molecular mechanisms between G2 and G3 regions, we performed pathway enrichment on the top 100 DEGs from the Visium analysis. While G2 tumors were enriched for the estrogen response early and late pathways, G3 tumors had enrichment only on estrogen response late pathway and more importantly, G3 had particular epithelial–mesenchymal transition (EMT) pathway enrichment supporting its poorly differentiated histological phenotype (Supplementary Fig. 3a/b). We then projected the top 20 DEGs identified in Visium clusters 2 and 4, along with lung epithelial marker EPCAM and NKX2-1, onto Xenium spatial clusters defined by Banksy. Seven of these DEGs were present in the Xenium 5K panel and showed distinct expression between cluster 10 (G2) and clusters 4/9 (G3), supporting biological consistency between platforms (Supplementary Fig. 3c).

### Tumor-of-origin in STAS derived from poorly differentiated LUAD are uncovered by Visium-HD and Xenium-5K

STAS is a risk factor for recurrence in early LUAD and may lead to an upstaging of the T category [6, 7]. Currently, no organoid or animal model has effectively replicated STAS, making human tissue-based studies of STAS in lung cancer even more important. In the LUAD sample, a STAS focus appeared in the lower bottom of the tissue, where disseminated tumor cells intermingle with numerous multinucleated giant cells (Fig. 1b). Geographically, these disseminated cells are closer to the moderately differentiated region, yet their cell-of-origin cannot be determined by morphology alone. Visium data indicated that the disseminated cancer cells in STAS were resulted from the poorly differentiated region (Cluster 2, Fig. 1b), in agreement with the prevailing concept that micropapillary tumors often drive STAS dissemination. Similarly, Xenium-derived tumor clusters also confirmed that the STAS foci originate from the G3 region (cluster4/9), even though they are physically closer to the G2 region (Fig. 1d right).

### Xenium surpasses Visium-HD in colorectal cancer cell segmentation

**Fig. 2:**
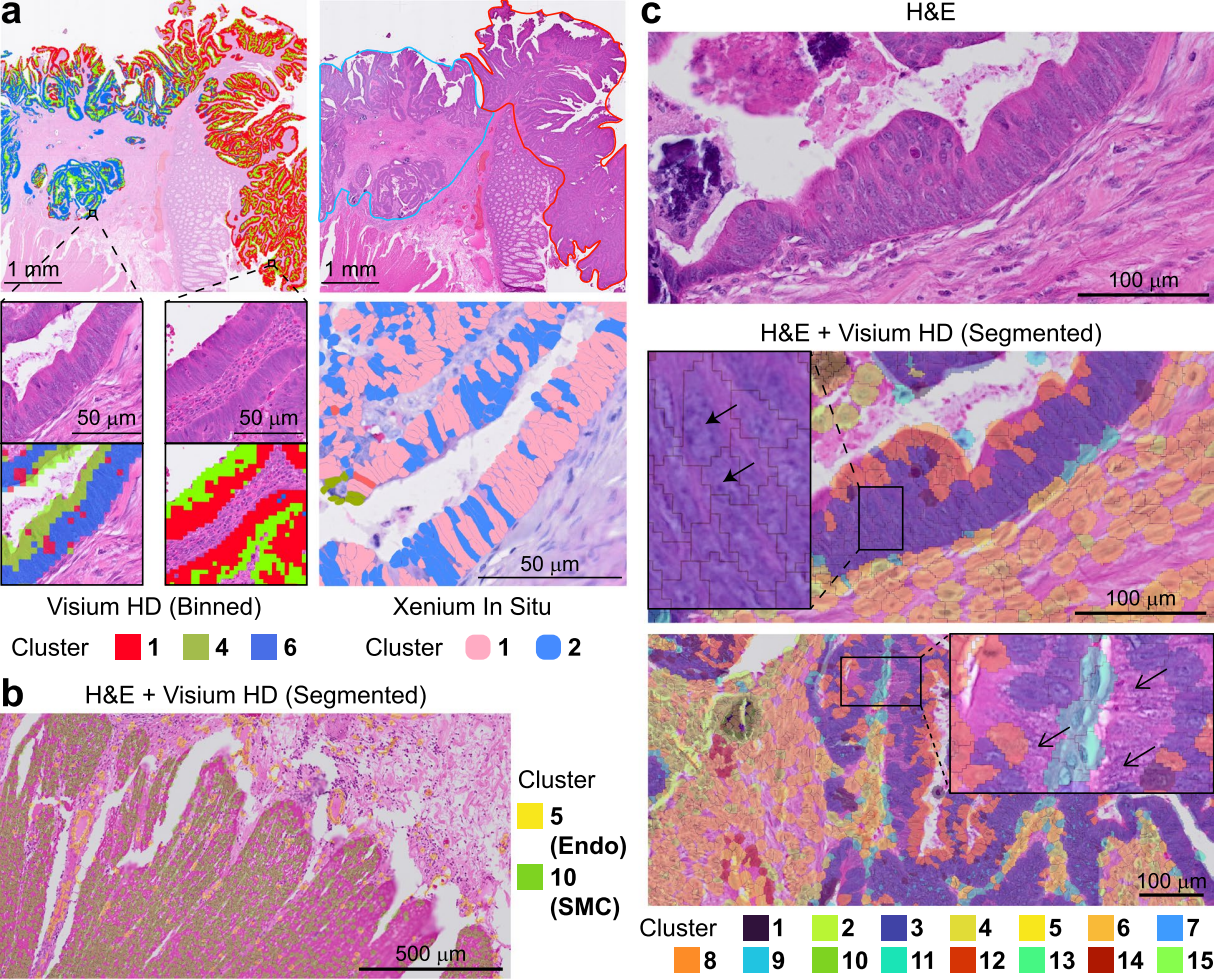
Cell segmentation between Visium-HD and Xenium In Situ in CRC data. **a** Left: Visium-HD, under 8 µm bins, splits tumor cells in both the invasive carcinoma and adenoma regions into separate basal and luminal fragments (clusters 1, 4, 6). Right: H&E image of the same colorectal section, with the adenoma outlined in red and the invasive carcinoma in blue. Xenium segmentation of the invasive region preserves the elongated, crowded, pseudostratified architecture of the tumor cells, matching the expected histology. **b** In normal colon region, single-cell re-segmentation of the Visium data successfully resolves endothelial (Endo) and smooth-muscle (SMC) cells at true single-cell resolution. **c** In colorectal-cancer regions, the segmented Visium data struggles with crowded, irregular nuclei: elongated tumor nuclei are frequently split, and some nuclei are missed entirely, causing reads from those areas to be discarded

We further analyzed a colorectal cancer (CRC) sample containing an adenoma or high-grade intraepithelial neoplasia (HGIN) region adjacent to an invasive adenocarcinoma region. In Visium-HD dataset, tumor cells from these two regions could be distinguished based on transcriptional profiles (Fig. 2a). However, due to the elongated morphology of colorectal tumor cells, the 8 µm bin-based segmentation used by Visium-HD often split a single cell into separate luminal and basal compartments. This segmentation artifact was observed in both the adenoma/HGIN and invasive areas, causing luminal segments from both tumor regions to cluster together due to similar transcriptomic identifies (Fig. 2a). This bin-level segmentation can complicate transcript-to-cell allocation and may hinder certain cross-region comparisons; nevertheless, Visium-HD still captures pronounced basal-side expression difference. Xenium dataset — generated from the same tissue — used a multimodal fluorescence-based cell segmentation approach and therefore maintained the structural integrity of elongated tumor cells (Fig. 2a). This approach preserves original cell boundaries from more irregularly shaped cells ensuring more accurate, one-to-one comparisons of gene expression between cells in the adenoma/HGIN and invasive regions. Recent efforts have optimized cell segmentation strategies for Visium HD data using the Bin2Cell and ENACT methodologies [8, 9]. 10 × Genomics has also added a segmentation module to the latest release of Space Ranger (see Methods). Using this workflow, we found that in morphologically well-organized regions of normal colon tissue, the algorithm achieved true single-cell resolution, allowing accurate segmentation and reliable identification of smooth-muscle and endothelial cells (Fig. 2b). By contrast, in tumor regions with densely packed or irregularly arranged nuclei, the H&E-based workflow still had inferior performance than Xenium’s morphology-guided approach: such as clusters of crowded cells were frequently unrecognized and discarded, and enlarge/irregular shaped cells were over-segmented (Fig. 2c). Collectively, these observations indicate that, while Visium’s current segmentation is adequate for well-structured tissues, limitations still exist in complex tissue complexes which may be compensated by Xenium.

### Technical comparison between ST in resolving delicate pulmonary and vascular micro-architectures

Normal lung alveoli are delicate structures composed of alveolar walls lined by a single layer of epithelial cells, primarily alveolar type 1 (AT1) and alveolar type 2 (AT2) cells, along with a rich capillary network in the interstitium [10]. In the Xenium 5K dataset, cells within a normal lung tissue field of view are classified into 23 distinct clusters. In contrast, in the Visium dataset, the same region is divided into 11 clusters, resulting in a lower histological resolution (Supplementary Fig. 4a). Differentiation between pneumocyte types is vital when researching lung injury and repair. In the Xenium dataset, AT1 and AT2 cells separated cleanly into clusters 18 and 20, respectively (Supplementary Fig. 4b). Differential-expression analysis confirmed mutually exclusive expression of canonical AT1 markers AGER and RTKN2 and the AT2 marker LAMP3 between two clusters (Supplementary Fig. 4b). By contrast, under 8 µm bins, using nuclei-based segmentation, Visium-HD struggled in alveolar niches failing to resolve AT1 from AT2 cells even after single-cell re-segmentation (Supplementary Fig. 4a right).

Another delicate structure relevant in cancer is the high endothelial venule (HEV), a specialized vessel with plump, cuboidal endothelial cells that facilitate lymphocyte homing to lymphoid tissues [11, 12]. HEVs often occur near dense lymphocyte accumulations and therefore precise cell segmentation for accurate detection and analysis is paramount [13]. In the LUAD sample, an HEV is found within a tertiary lymphoid structure. In the Xenium dataset, HEV endothelial cells are defined as cluster 13 (green colored), whereas in the Visium-HD dataset, the HEV area is largely allocated into cluster 1 (Supplementary Fig. 4c). When aligned with H&E images, we confirmed that cluster 1 in Visium rather represents a “catch-all” group containing un-segmented cell types, stromal voids or empty background with low UMI counts.

### Technical comparison between ST in pigmented cell regions

In the LUAD sample, dust cells were observed, which are large, round cells with a foamy, granular cytoplasm containing ingested particles, often dust, giving them a dark appearance in the HE image [14]. Xenium relies on fluorescent detection probably interfering with light-directed detection in those areas even though cell contours can be preserved with nuclei staining (Supplementary Fig. 4 d). As the technical counterpart, Visium is less affected generating decent reads within those regions (Supplementary Fig. 4 d). In practice, cancers including melanoma, pigmented basal cell carcinoma, and pigmented squamous cell carcinoma have those pigmented regions so cares must be taken when interpretating results.

## Discussion

A side-by-side comparison underscores the strengths and limitations of the two ST technologies. While Visium-HD is highly adept at elucidating molecular differences between tumors with varied differentiation status, Xenium achieves sub-cellular resolution and retains fine histological details, making it “histology-friendly” for spatial analyses. Researchers should choose appropriate STs and design experiments to address specific questions in need, considering factors such as the depth for pathological information, balance between detection sensitivity and gene coverage, sample-to-region cost, and the tissue types under investigation that summarized in Supplementary Table 2. Due to limited samples under analysis, our conclusion may still be preliminary and with the continual accumulation of difference ST datasets, technical improvement and computational advances in ST data processing, our technical remarks will be updated.

## Supplementary Information

Supplementary Material 1: Supplementary Fig. 1. Transcript-level detection comparison between Visium-HD and Xenium-5K in the same LUAD slide. (a) Pie chart of detected genes showing that 13 254 genes are unique to Visium-HD (72.6%), 173 are unique to Xenium-5K (0.9%), and 4,828 are detected by both platforms (26.4%). (b) Logscale comparison between the raw counts of the overlapping genes in two STs (*n* = 4,828, *r* = 0.64, *p* < 1 × 10^-5^). (c) Empirical cumulative distribution functions of Xenium-5K counts indicate similar abundance profiles for overlap genes and Xenium-only genes. (d) Stacked barplots summarizing cumulative raw-counts by gene group for each platform. Visium-only genes dominate the Visium-HD library, whereas overlap genes account for the majority of Xenium-5K reads.

Supplementary Material 2: Supplementary Fig. 2. Spatial distribution of ILDR2 and ABCC6 transcripts on the same LUAD section. The top panel plots ILDR2 transcripts (green dots)—a gene detected by both Visium-HD and Xenium-5K—across the tissue; tumor-grade contours are colored blue (G3), yellow (G2) and green (normal lung). The bottom panel shows distribution of ABCC6 transcripts (magenta dots) in different regions of LUAD slide, which were captured only by Xenium-5K.

Supplementary Material 3: Supplementary Fig. 3. Differential-expression analysis between grade 2 and grade 3 LUAD tumors. (a) Hallmark pathway enrichment for genes up-regulated in grade 2 tumors detected in Visium. (b) Hallmark pathway enrichment for genes up-regulated in grade 3 tumors in Visium. (c) Dot-plot showing expression of selected DEGs, together with the epithelial markers NKX2-1 and EPCAM, across Banksy-defined cell clusters.

Supplementary Material 4: Supplementary Fig. 4. Comparison of Visium-HD and Xenium-5K performance in analyzing irregular structures and pigmented cells. (a) Side-by-side H&E, Xenium-5K, Visium-HD, and single-cell–re-segmented Visium-HD views of the same normal-lung field, with color-coded clusters. (b) Overlay of H&E and Xenium-5K data highlighting AT1 and AT2 cells. Heatmap shows differentially expressed genes between clusters 18 (AT1) and 20 (AT2). (c) Overlay of H&E and Xenium-5K with high endothelial venule (HEV) colored in green. Visium-HD of the same region is shown in the right with colored individual clusters. (d) Spatial transcript-density maps of a dust-cell–rich (pigmented macrophage) area rendered with Visium-HD and Xenium-5K.

Supplementary Material 5: Supplementary Table 1. TOP 100 differentially expressed genes identified between Cluster 2 (corresponding to Grade 3 tumor) and Cluster 4 (corresponding to Grade 2 tumor) in LUAD analyzed with Visium-HD.

Supplementary Material 6: Supplementary Table 2. Practical comparison of Xenium 5K and Visium HD ST platforms.

Supplementary Material 7.

## Data Availability

Please see our Online Methods for data sources. Part of our data was downloaded from reference 1 of this article, which is now published in Nature Genetics: https://www.nature.com/articles/s41588-025-02193-3.

## Abbreviations

ST: Spatial transcriptomics
LUAD: Invasive lung adenocarcinoma
STAS: Single focus of spreading through airspace
DEG: Differentially expressed gene
EMT: Epithelial–mesenchymal transition
CRC: Colorectal cancer
HGIN: High-grade intraepithelial neoplasia
AT: Alveolar type
HEV: High endothelial venule
