# Correction: Comparing Xenium 5K and Visium HD data from identical tissue slide at a pathological perspective

**DOI:** 10.1186/s13046-026-03775-7

**Published:** 2026-07-16

**Authors:** Mengping Long, Taobo Hu, Weixin Wang, Junshun Gao, Nan Wang, Mats Nilsson

**Affiliations:** 1 Science for Life Laboratory, Department of Biochemistry and Biophysics, Stockholm University, Stockholm, Sweden; 2 Cosmos Wisdom Biotech Co. Ltd, Building 10th, No. 617 Jiner Road, Hangzhou, 311215 China

**Correction: J Exp Clin Cancer Res 44, 219 (2025)**

**https://doi.org/10.1186/s13046-025-03479-4**

Following the publication of the original article [1], the authors have minor corrections in the Introduction section and in the Fig. 2 image and caption. The details are below:

**Introduction**

Replace “on the identical lung and colon tissues”with“on the identical lung tissue”

**Figure 2 image**

The CRC dataset used in Fig. 2 is a Xenium In Situ dataset, not a Xenium 5K dataset. Replace “Xenium 5K” to “Xenium In Situ”

**Figure 2 caption**

Replace “Cell segmentation between Visium-HD and Xenium-5K in CRC data.” with “Cell segmentation between Visium-HD and Xenium In Situ in CRC data.”

The corrections do not compromise the validity of the conclusions and the overall content of the article. The original article [1] has been corrected.
