# Supplementary material for: Comparing Xenium 5K and Visium HD data from identical tissue slide at a pathological perspective
Source: J Exp Clin Cancer Res. 2025 Jul 26;44:219. doi: 10.1186/s13046-025-03479-4 (PMC12298044; doi:10.1186/s13046-025-03479-4)
Supplement: Supplementary file 1 — Supplementary Material 1: Supplementary Fig. 1. Transcript-level detection comparison between Visium-HD and Xenium-5K in the same LUAD slide. (a) Pie chart of detected genes showing that 13 254 genes are unique to Visium-HD (72.6%), 173 are unique to Xenium-5K (0.9%), and 4,828 are detected by both platforms (26.4%). (b) Logscale comparison between the raw counts of the overlapping genes in two STs (n = 4,828, r = 0.64, p < 1 × 10^-5^). (c) Empirical cumulative distribution functions of Xenium-5K counts indicate similar abundance profiles for overlap genes and Xenium-only genes. (d) Stacked barplots summarizing cumulative raw-counts by gene group for each platform. Visium-only genes dominate the Visium-HD library, whereas overlap genes account for the majority of Xenium-5K reads. [file 13046_2025_3479_MOESM1_ESM.pdf]

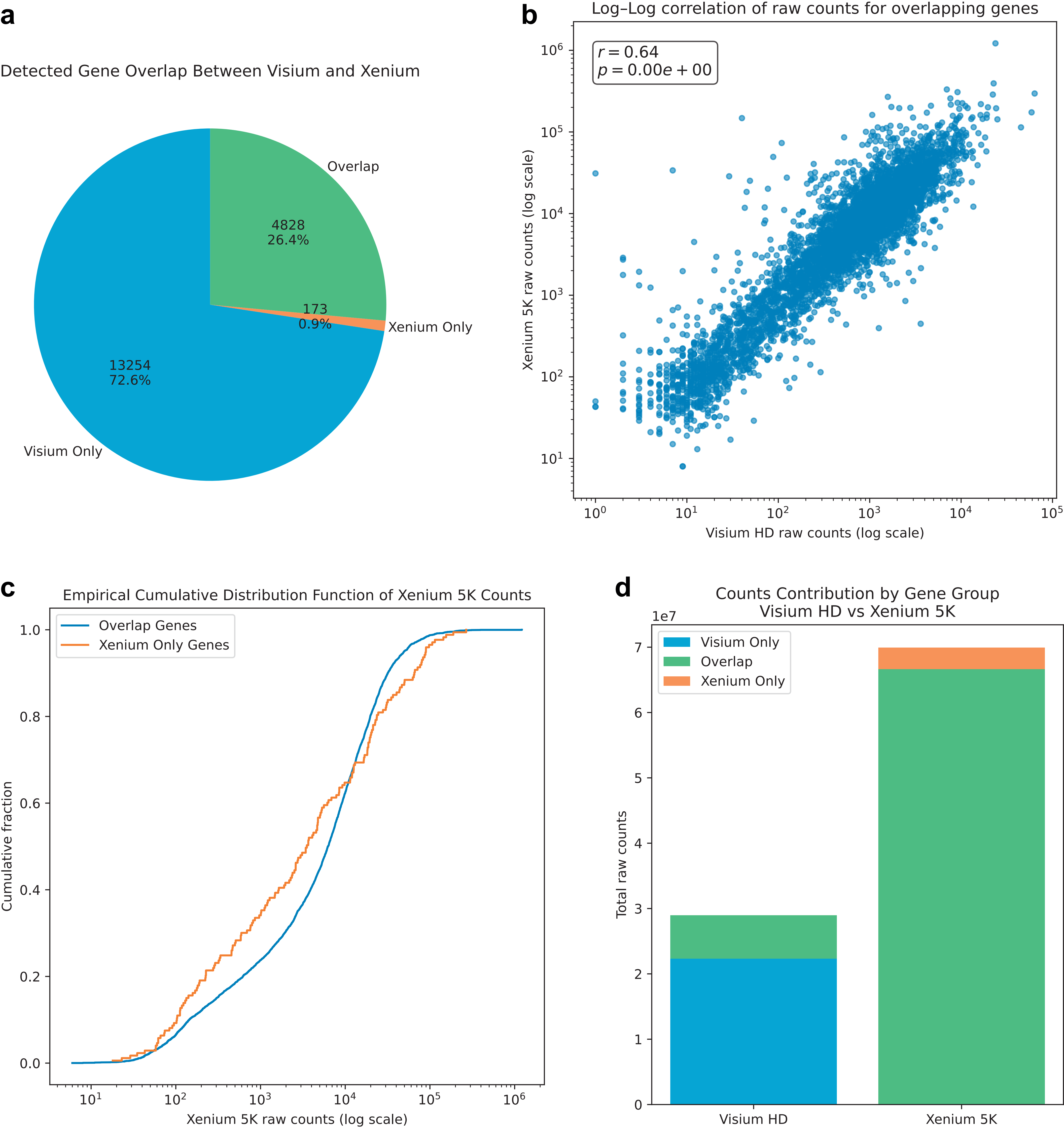
a
Detected Gene Overlap Between Visium and Xenium
Overlap
4828
26.4%
173
0.9%
Xenium Only
13254
72.6%
Visium Only
b
Log–Log correlation of raw counts for overlapping genes
$r = 0.64$
$p = 0.00e+00$
Xenium 5K raw counts (log scale)
Visium HD raw counts (log scale)
c
Empirical Cumulative Distribution Function of Xenium 5K Counts
Overlap Genes
Xenium Only Genes
Cumulative fraction
Xenium 5K raw counts (log scale)
d
Counts Contribution by Gene Group
Visium HD vs Xenium 5K
1e7
Visium Only
Overlap
Xenium Only
Total raw counts
Visium HD
Xenium 5K
