# Supplementary figures and images for: Comparing Xenium 5K and Visium HD data from identical tissue slide at a pathological perspective
Source: J Exp Clin Cancer Res. 2025 Jul 26;44:219. doi: 10.1186/s13046-025-03479-4 (PMC12298044; doi:10.1186/s13046-025-03479-4)

Xenium 5K & Visium HD Overlap Gene

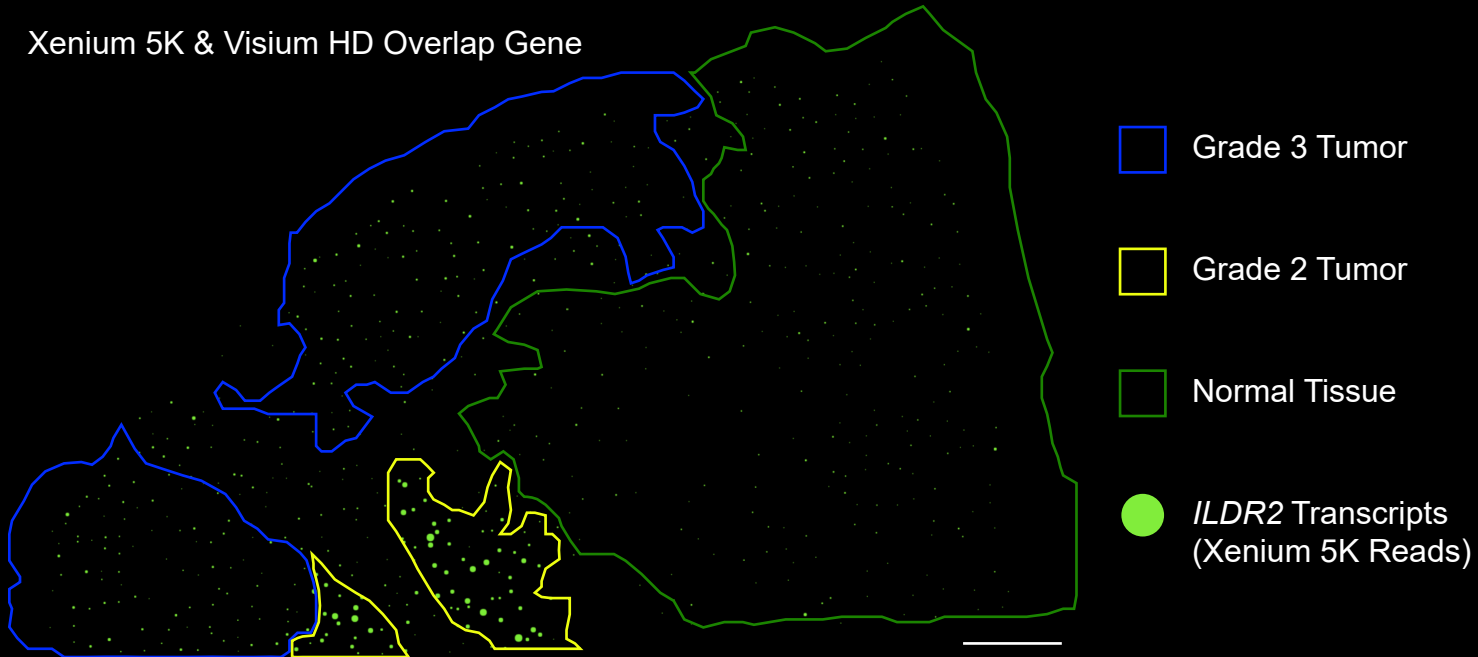

Xenium 5K Only Gene

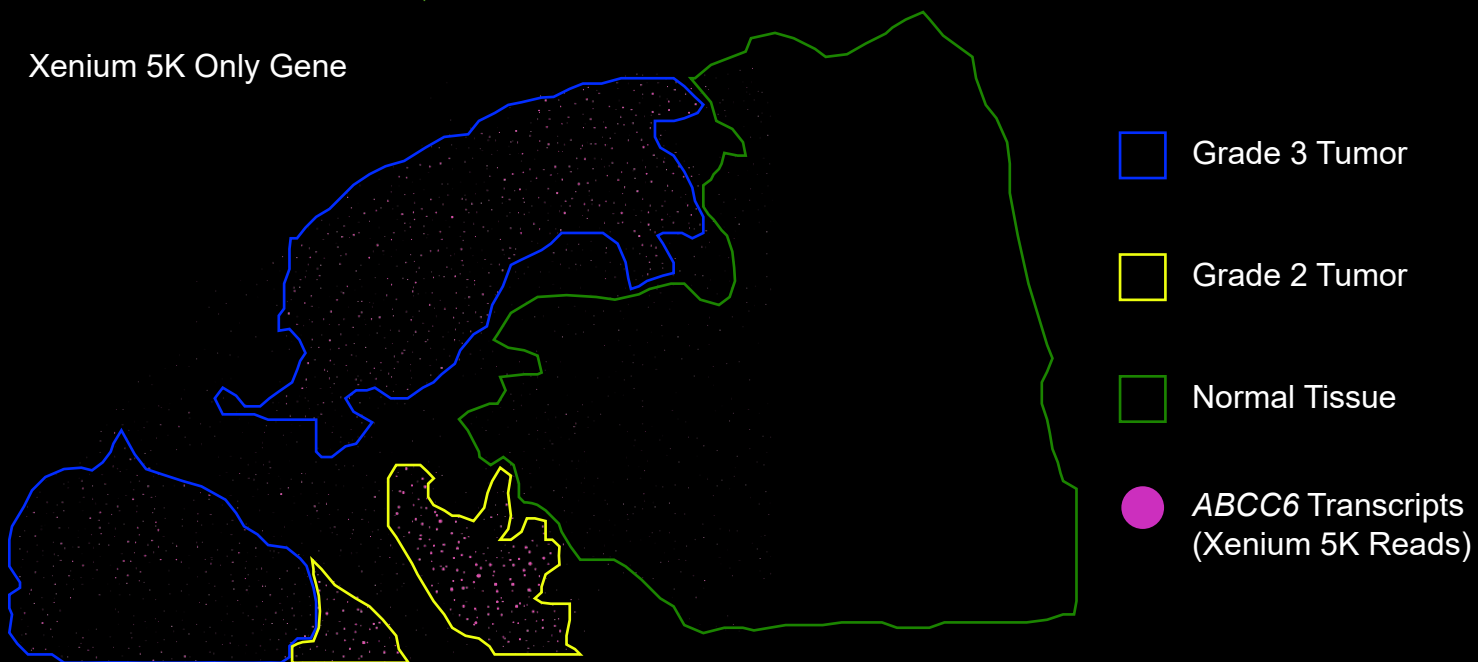

Supplement: Supplementary file 2 — Supplementary Material 2: Supplementary Fig. 2. Spatial distribution of ILDR2 and ABCC6 transcripts on the same LUAD section. The top panel plots ILDR2 transcripts (green dots)—a gene detected by both Visium-HD and Xenium-5K—across the tissue; tumor-grade contours are colored blue (G3), yellow (G2) and green (normal lung). The bottom panel shows distribution of ABCC6 transcripts (magenta dots) in different regions of LUAD slide, which were captured only by Xenium-5K. [file 13046_2025_3479_MOESM2_ESM.pdf]

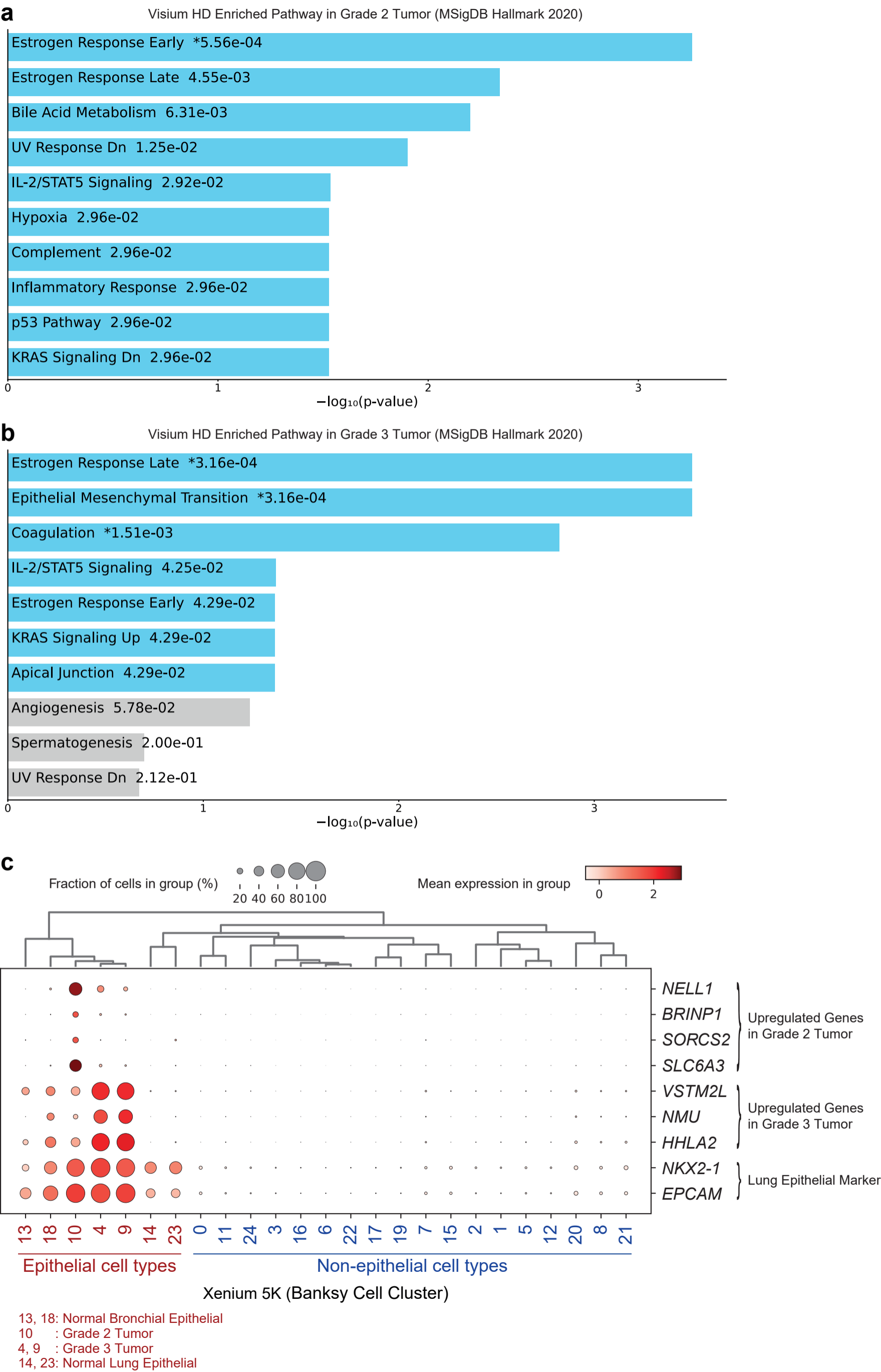

Supplement: Supplementary file 3 — Supplementary Material 3: Supplementary Fig. 3. Differential-expression analysis between grade 2 and grade 3 LUAD tumors. (a) Hallmark pathway enrichment for genes up-regulated in grade 2 tumors detected in Visium. (b) Hallmark pathway enrichment for genes up-regulated in grade 3 tumors in Visium. (c) Dot-plot showing expression of selected DEGs, together with the epithelial markers NKX2-1 and EPCAM, across Banksy-defined cell clusters. [file 13046_2025_3479_MOESM3_ESM.pdf]

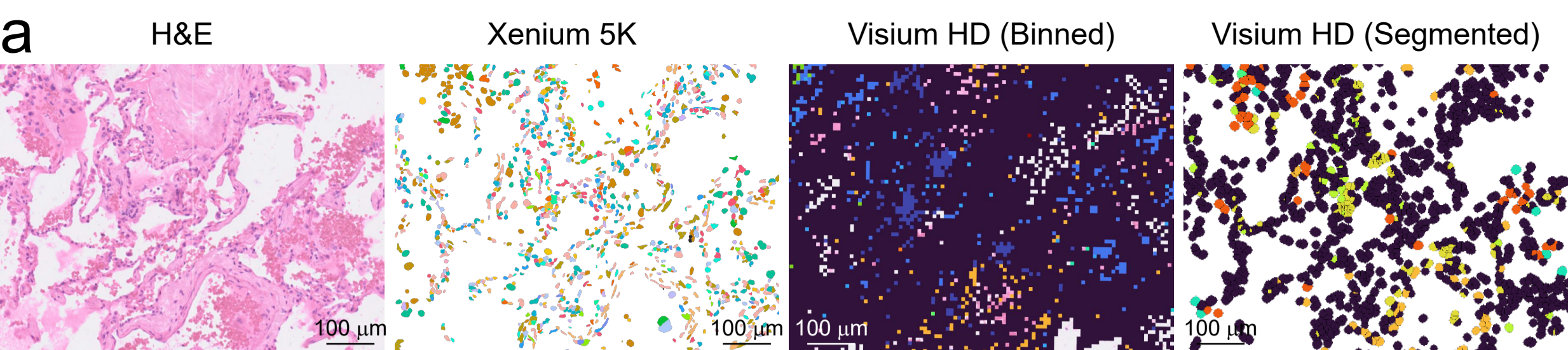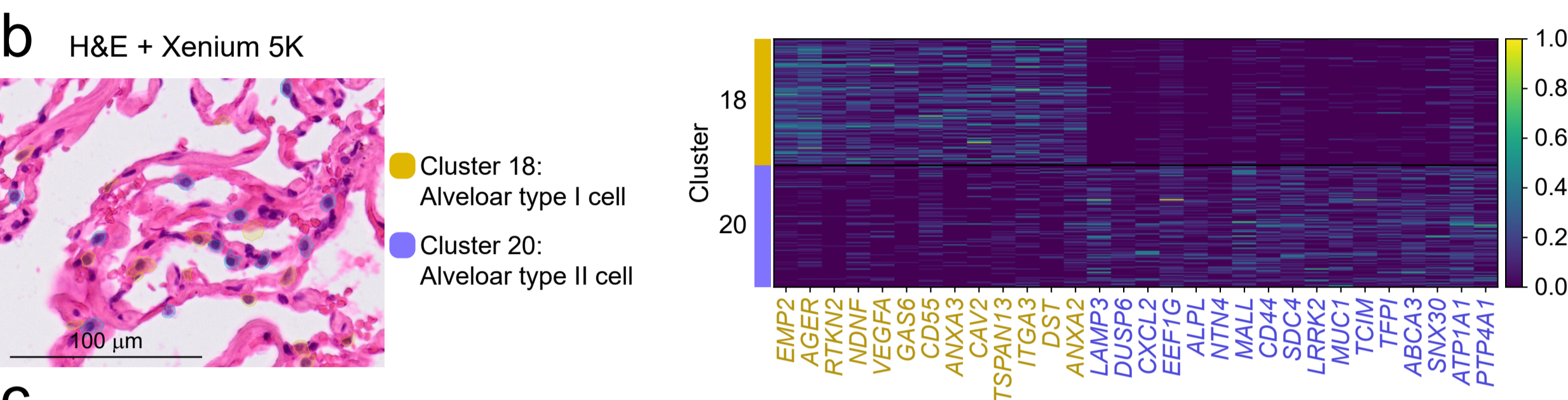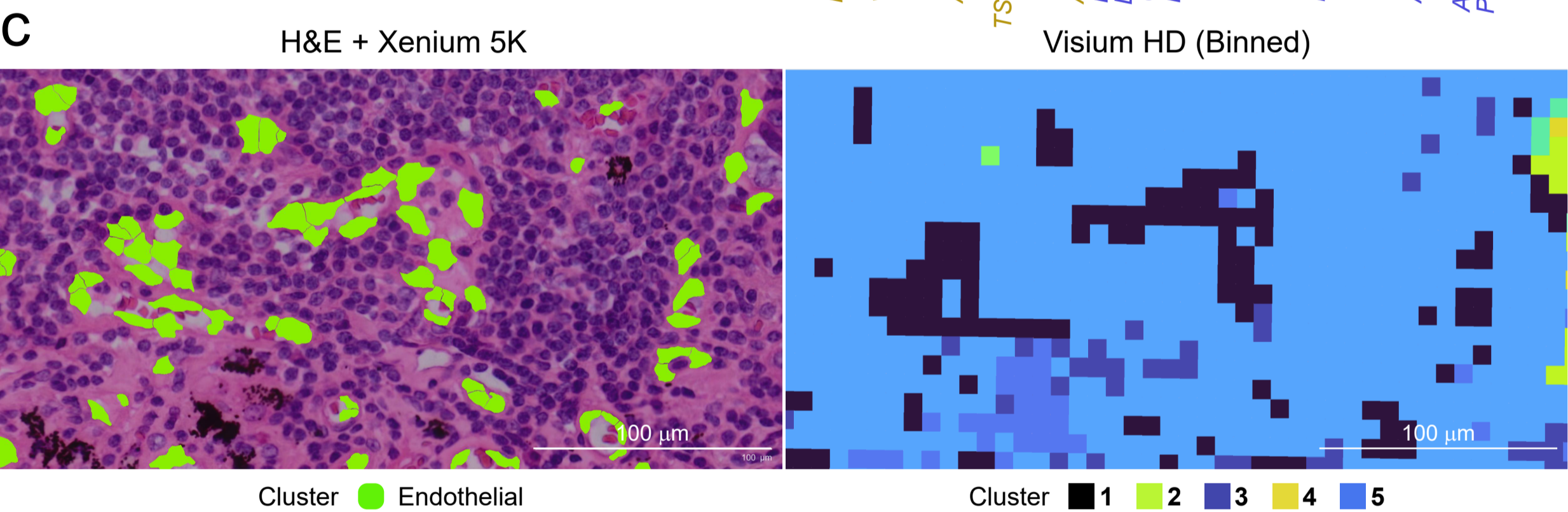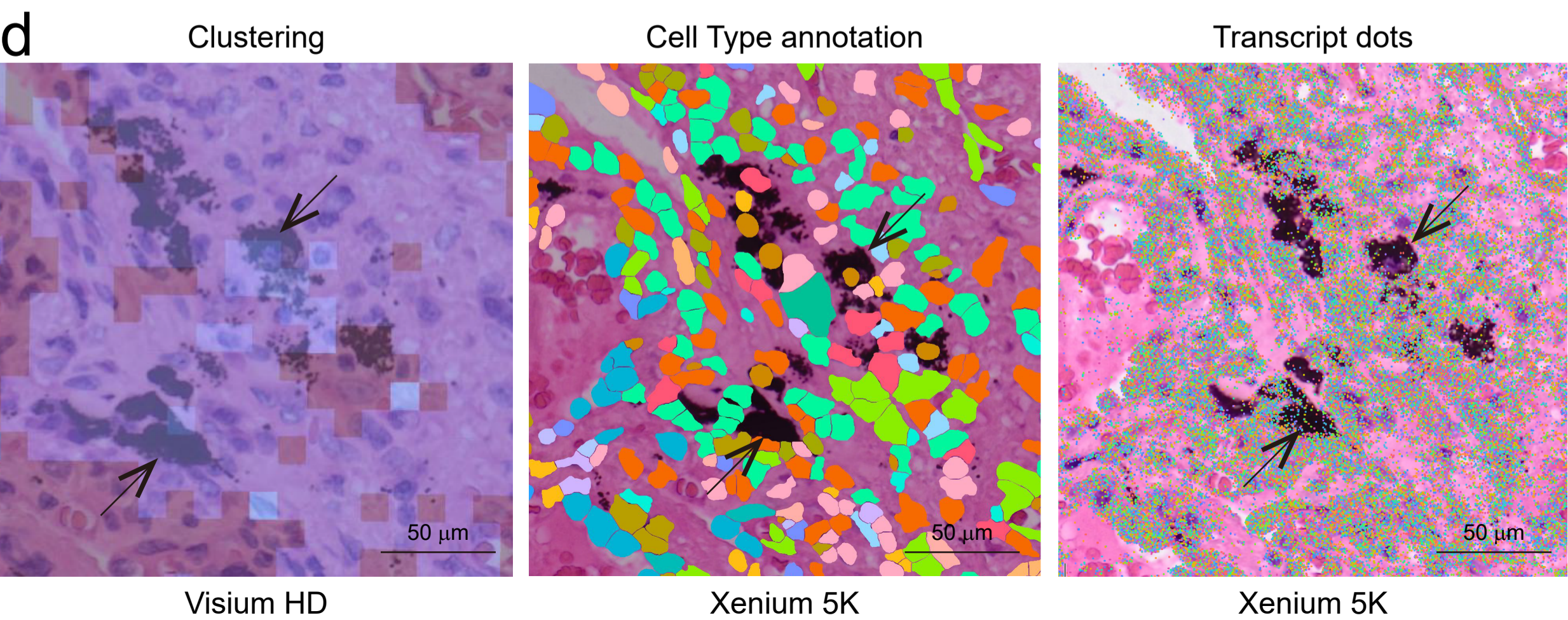

Supplement: Supplementary file 4 — Supplementary Material 4: Supplementary Fig. 4. Comparison of Visium-HD and Xenium-5K performance in analyzing irregular structures and pigmented cells. (a) Side-by-side H&E, Xenium-5K, Visium-HD, and single-cell–re-segmented Visium-HD views of the same normal-lung field, with color-coded clusters. (b) Overlay of H&E and Xenium-5K data highlighting AT1 and AT2 cells. Heatmap shows differentially expressed genes between clusters 18 (AT1) and 20 (AT2). (c) Overlay of H&E and Xenium-5K with high endothelial venule (HEV) colored in green. Visium-HD of the same region is shown in the right with colored individual clusters. (d) Spatial transcript-density maps of a dust-cell–rich (pigmented macrophage) area rendered with Visium-HD and Xenium-5K. [file 13046_2025_3479_MOESM4_ESM.pdf]
