# Supplementary material for: Comparing Xenium 5K and Visium HD data from identical tissue slide at a pathological perspective
Source: J Exp Clin Cancer Res. 2025 Jul 26;44:219. doi: 10.1186/s13046-025-03479-4 (PMC12298044; doi:10.1186/s13046-025-03479-4)
Supplement: Supplementary file 6 — Supplementary Material 6: Supplementary Table 2. Practical comparison of Xenium 5K and Visium HD ST platforms. [file 13046_2025_3479_MOESM6_ESM.docx]

Supplementary Table 2. Practical comparison of Xenium 5K and Visium HD spatial-transcriptomics platforms

|  | **Xenium 5K** | **Visium HD** | **Technical notes** |
| --- | --- | --- | --- |
| Ease of use for tissue preparation | Need designated glass slides | Universal pathological slides |  |
| Guide costs per 0.5mmX0.5mm area | 2.4K USD | 3.8K USD | HD includes sequencing cost |
| Typical sample capacity range per assay unit | 1-18 | 1-4 | Based on experience |
| Compatibility for downstream multiplex fluorescence | Easy | Challenging |  |
