# Supplementary material for: Comparing Xenium 5K and Visium HD data from identical tissue slide at a pathological perspective
Source: J Exp Clin Cancer Res. 2025 Jul 26;44:219. doi: 10.1186/s13046-025-03479-4 (PMC12298044; doi:10.1186/s13046-025-03479-4)
Supplement: Supplementary file 7 — Supplementary Material 7. [file 13046_2025_3479_MOESM7_ESM.docx]

**Online Methods**

**Image Segmentation Strategies**

Xenium-5K uses multi-modal segmentation. DAPI staining is used for nuclear staining and nucleus segmentation, while antibodies for ATP1A1, E-Cadherin, and CD45 are employed to delineate a broad range of cell membrane boundaries. Additionally, 18S ribosomal RNA labels the cytoplasm and serves as a pan-cell type marker. The cocktail also includes alpha-SMA and Vimentin antibodies for intracellular protein staining. Visium-HD captures transcript expression in a continuous grid of 2x2 µm squares. The default analysis pipeline starts with an 8 µm pin resolution to ensure sufficient transcript capture per bin. We use this as a default input for clustering analysis without cell annotation.

**Cell Clustering**

For clustering, principally, a sparse nearest-neighbor graph is first constructed by connecting each spot to its k nearest neighbors, where k is scaled logarithmically with the total number of spots. Louvain Modularity Optimization is then applied to identify densely connected modules in the graph. Hierarchical clustering on the cluster-medoids in PCA space is performed, and pairs of sibling clusters are merged if no differentially expressed genes (adjusted p-value < 0.05) are found. This merging step is repeated until no further clusters can be combined. Banksy is a graph-based clustering method applied in single-cell or spatial transcriptomics to identify distinct cell populations. It constructs a k-nearest neighbors graph, and then uses a community-detection algorithm (by default Leiden) controlled by parameters like resolution. By tuning parameters such as k, lambda, and resolution, it reveals biologically meaningful clusters while minimizing over- or under-clustering. Key parameters for the Banksy algorithm we applies lambda set to 0, a resolution of 0.9, k (nearest neighbors) = 50, and the use of Leiden clustering.
**Cell-Level Binning of Visium HD**High-resolution Visium HD spatial transcriptomic libraries from multiple FFPE tissue sections are processed with Space Ranger v4.0.1 (10x Genomics released in June 2025). For every sample, raw FASTQ archives are unpacked and the accompanying bright-field .btf image was converted to OME-TIFF with Bio-Formats CLI (bfconvert) v6.11.0 running under OpenJDK 17. The count workflow iss executed in local mode on a 16-core, 128 GB RAM workstation, supplying the GRCh38-2024-A reference transcriptome, the Visium Human Transcriptome Probe Set v2.0 (GRCh38-2020-A), relevant slide serial and capture-area identifiers, high-resolution H & E and CytAssist images, and using `--create-bam=false` to suppress BAM output. During processing, reads are aligned, tissue-overlapping 2 µm barcodes are filtered, and nuclei are segmented with the built-in StarDist v6 model. Each nucleus mask is expanded by the default 15 µm radius, and all underlying barcodes are aggregated into single-cell bins, yielding gene-by-cell count matrices along with standard 8 µm and 16 µm binned matrices, segmentation masks, and an HTML quality-control report for downstream analyses.
**Data Source**

All data used in this study were obtained from the 10x Genomics website. The Visium HD lung cancer dataset, titled Visium_HD_Human_Lung_Cancer_post_Xenium_Prime_5K_Experiment2, was downloaded from https://www.10xgenomics.com/datasets/visium-hd-cytassist-gene-expression-human-lung-cancer-post-xenium-expt. The corresponding Xenium dataset, Xenium_Prime_Human_Lung_Cancer_FFPE_outs, was sourced from https://www.10xgenomics.com/datasets/xenium-human-lung-cancer-post-xenium-technote. For colon cancer, the Visium HD dataset (Visium HD, Sample P2 CRC) and the Xenium In Situ dataset (Xenium In Situ, Sample P2 CRC) were both obtained from <https://www.10xgenomics.com/platforms/visium/product-family/dataset-human-crc.>

**Code and Data Availability**

All custom analysis scripts developed by the authors for this study have been deposited in the public repository <https://github.com/hutaobo/Xenium_5K_Visium_HD_Compare.> The Visium HD datasets processed with Space Ranger (segmented outputs) have been archived on Zenodo: https://zenodo.org/uploads/15722536.
